# Cost-effectiveness analysis for joint pain treatment in patients with osteoarthritis treated at the Instituto Mexicano del Seguro Social (IMSS): Comparison of nonsteroidal anti-inflammatory drugs (NSAIDs) vs. cyclooxygenase-2 selective inhibitors

**DOI:** 10.1186/1478-7547-6-21

**Published:** 2008-11-12

**Authors:** Iris Contreras-Hernández, Joaquín F Mould-Quevedo, Rubén Torres-González, María Victoria Goycochea-Robles, Reyna Lizette Pacheco-Domínguez, Sergio Sánchez-García, Juan Manuel Mejía-Aranguré, Juan Garduño-Espinosa

**Affiliations:** 1 Unidad de Investigación en Economía de la Salud, Instituto Mexicano del Seguro Social, Mexico, D.F, Mexico; 2 Hospital de Traumatología y Ortopedia: Unidad Médica de Alta Especialidad "Dr. Victorio de la Fuente Narváez", Instituto Mexicano del Seguro Social, Mexico, D.F, Mexico; 3 Hospital General Regional No. 1, Instituto Mexicano del Seguro Social, Mexico, D.F, Mexico; 4 Unidad de Investigación en Servicios de Salud, Envejecimiento, Instituto Mexicano del Seguro Social, Mexico, D.F, Mexico; 5 Unidad de Investigación en Epidemiología Clínica, UMAE Hospital de Pediatría, Instituto Mexicano del Seguro Social, Mexico, D.F, Mexico

## Abstract

**Background:**

Osteoarthritis (OA) is one of the main causes of disability worldwide, especially in persons >55 years of age. Currently, controversy remains about the best therapeutic alternative for this disease when evaluated from a cost-effectiveness viewpoint. For Social Security Institutions in developing countries, it is very important to assess what drugs may decrease the subsequent use of medical care resources, considering their adverse events that are known to have a significant increase in medical care costs of patients with OA. Three treatment alternatives were compared: celecoxib (200 mg twice daily), non-selective NSAIDs (naproxen, 500 mg twice daily; diclofenac, 100 mg twice daily; and piroxicam, 20 mg/day) and acetaminophen, 1000 mg twice daily. The aim of this study was to identify the most cost-effective first-choice pharmacological treatment for the control of joint pain secondary to OA in patients treated at the Instituto Mexicano del Seguro Social (IMSS).

**Methods:**

A cost-effectiveness assessment was carried out. A systematic review of the literature was performed to obtain transition probabilities. In order to evaluate analysis robustness, one-way and probabilistic sensitivity analyses were conducted. Estimations were done for a 6-month period.

**Results:**

Treatment demonstrating the best cost-effectiveness results [lowest cost-effectiveness ratio $17.5 pesos/patient ($1.75 USD)] was celecoxib. According to the one-way sensitivity analysis, celecoxib would need to markedly decrease its effectiveness in order for it to not be the optimal treatment option. In the probabilistic analysis, both in the construction of the acceptability curves and in the estimation of net economic benefits, the most cost-effective option was celecoxib.

**Conclusion:**

From a Mexican institutional perspective and probably in other Social Security Institutions in similar developing countries, the most cost-effective option for treatment of knee and/or hip OA would be celecoxib.

## Background

Osteoarthritis (OA) is a progressive disorder characterized by the destruction of joint cartilage and subchondral bone, as well as changes in the synovium [1]. Worldwide, it is one of the most important causes of disability. OA ranks 4th as a disabling disease in women and ranks 8th in men [1,2]. OA is the most frequent joint disease. Because the knee is a weight-bearing joint, it is the most affected; ~10% of the population suffering from knee OA has disabling symptomatology [3].

The main objectives of OA pharmacotherapy are to achieve an anti-inflammatory and analgesic effect [4,5]. Analgesic and anti-inflammatory properties of nonsteroidal anti-inflammatory drugs (NSAIDs) are based on the inhibition of the cyclooxygenase (COX) enzyme isoforms [6]. Traditional NSAIDs inhibit both isoforms of the COX enzyme responsible for the first step in the conversion of arachidonic acid into a variety of prostaglandins, thromboxanes and leukotrienes in the body [7]. Anti-inflammation and pain decrease with the effects of NSAIDs, resulting from the inhibition of COX-2-mediated prostaglandin synthesis at the site of the damaged tissue, whereas gastrointestinal (GI) complications are due to the inhibition of COX-1-mediated prostaglandin synthesis in the GI mucosa. Therefore, it was assumed that COX-2 inhibitors should treat pain but without gastric toxicity [7]. Nevertheless, COX-2 inhibitors have also been associated with risk of GI toxicity, but the most noticeable risks are those associated with cardiovascular diseases and renal toxicity [8,9].

However, these effects have shown to be dose-dependent and a class effect has not been reported. Celecoxib, at a dose of 200 mg/day or less, has similar or fewer risks than those observed for the traditional NSAIDs [6,9,10]. Acetaminophen has few risks for cardiovascular or renal complications, although it has a higher risk for liver complications [4]. In addition, this drug has the lowest rate for decreasing inflammation [11,12]. Drugs such as naproxen and ibuprofen have a higher analgesic and anti-inflammatory effect, but the risk of GI bleeding is increased, events that markedly increase medical care costs [8]. These drugs carry a certain risk for cardiovascular disorders; however, it is not unacceptable, especially with the use of naproxen [13].

When NSAIDs such as naproxen and ibuprofen were compared to coxibs, it was observed that both drugs significantly decreased pain in percentages similar to those observed in patients randomized to choice of drug; however, differences were noteworthy in regard to coxibs with shorter time until pain relief as well as the control of dyspeptic-type GI complications in up to 15% [14] and up to 50% in peptic ulcer perforation-like GI complications [15,16]. All this led the American Pain Society to place coxibs as the first-choice drugs for the initial treatment of joint pain in OA regardless of its higher cost as compared to nonselective NSAIDs [17].

Some economic evaluation studies already published have attempted to estimate OA care costs. In a study published in the U.S. in 1998, it is mentioned that medical care costs for this disease, from the viewpoint of service suppliers, range from $5000 to $6000 dollars/patient-years, depending on patient age and on disease evolution [18]. In Canada, a study was published that reported mean annual health care costs per patient for 1999 and these were estimated at $2456 Canadian dollars [19]. Moreover, in Italy, in a 1-year study, mean cost per patient was estimated only considering direct costs at~934 Euros [20]. An important cause for the increase in health care costs for patients with OA is the treatment of adverse events associated with the use of nonselective NSAIDs. It has been consistently documented that medical consultations, need for hospitalizations and, many times, the use of concomitant drugs are increased. Therefore, from a cost-effectiveness viewpoint, the use of coxibs in this group of patients is very attractive. It was observed that the use of coxibs, instead of nonselective NSAIDs, in the group of patients with high-risk OA, substantially decreased incremental cost-effectiveness ratio from $275,809 USD to $55,803 USD per each QALY (quality-adjusted life years) saved [21].

In a developing country such as Mexico with low resources for drug acquisition and care for drug-related complications [22,23], it is essential to conduct cost-effectiveness assessment not only to compare costs of two or three drugs, but also to evaluate drug side effects. It is important to point out that the IMSS covers the health needs of 39% of the Mexican population [23] and is divided into three levels of health care: (1) family medical care; (2) specialist care and hospitalization, and (3) difficult-to-control and complex diseases that demand a higher degree of medical specialization [24,25].

IMSS uses acetaminophen, nonselective NSAIDs (diclofenac, naproxen, piroxicam), and celecoxib (cyclooxygenase-2 inhibitor) for the treatment of pain due to OA. Thus, the primary consumer of the information will be the Institution itself through its operational staff. A description of medical practice based on the use of resources for treatment of OA and its cost within the same Institution will be performed. Currently, these types of evaluations are a priority for Social Security institutions in developing countries [26].

The objective of this study was to identify the most cost-effective, first-choice pharmacological treatment for the control of joint pain secondary to OA of the knee and/or hip in patients treated at the IMSS.

## Methods

### Decision Tree Model

The study constructed an analytical model that may reproduce and simplify the clinical reality observed in patients with OA treated with alternatives compared with the treatment of joint pain at the IMSS. The proposed model aimed to identify the probability to control pain among the different therapies as well as the potential development of GI, renal and/or cardiovascular complications during a 6-month time horizon. The clinical significance of adverse drug events leads us to recognize them as an acute situation. When they occur, an action is generated. In the case of study alternatives, the action may be to administer concomitant treatment or drug discontinuation. In this way, none of these events should occur again in the same subject. Thus, it is not possible to describe the phenomenon as a series of observational cycles but as a group of events occurring as one being a consequence of another. Therefore, we considered that the best descriptive analysis was the decision tree model.

**Figure 1:**
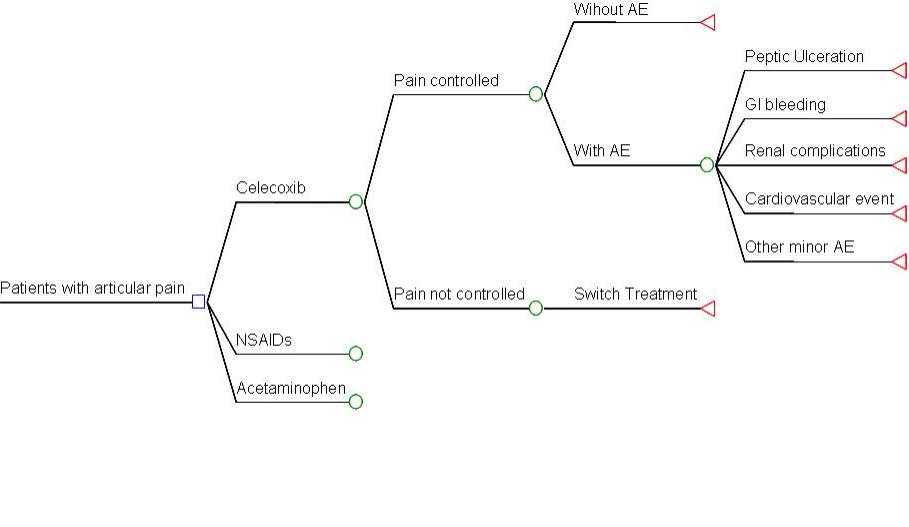
**Decision tree.** Reproduction of clinical reality observed in patients with osteoarthritis (OA) receiving one of the alternatives to be compared for the treatment of joint pain, found in each of the three health care levels at the Instituto Mexicano del Seguro Social, identifying the probability to control pain, as well as the development of gastrointestinal, renal and/or cardiovascular complications. NSAIDs, nonsteroidal anti-inflammatory drugs; GI, gastrointestinal.

The model starts with the description of a base case of an adult patient diagnosed with OA of the knee and/or hip and the need for pharmacological treatment for severe joint pain. Three decision nodes corresponding to the three alternatives (acetaminofen, nonselective NSAIDs or celecoxib) are generated. The first probabilistic node corresponding to pain improvement or no pain improvement arises from each of them. The "no improvement" branch corresponds to therapeutic failure and the prescription of one of the two remaining alternatives available is mandatory, with a new generation of branches, pain control or no pain control. From the latter, another branch arises now using the remaining treatment option. The next tree branch, as a consequence of pain improvement, is divided into the presence of adverse effects or no adverse effects. When no adverse events occur, it is converted into a terminal node and is considered a therapeutic success, thus corresponding to the effectiveness measure. The next probabilistic node arises from the occurrence of adverse events towards the probability for the development of gastric symptoms, GI bleeding, renal toxicity, and cardiovascular events during a 6-month period of continuous treatment with these drugs. A schematic flow chart is shown in Figure 1.

### Medication

Considering the scenario of a patient with severe joint pain secondary to knee and/or hip OA, a comparison of the costs generated by medical care of patients with OA receiving any of the three possible treatment alternatives was proposed; these alternatives were based on IMSS Drug Formulary and international guidelines [4,27]: celecoxib, 200 mg twice daily; non-selective NSAIDs (naproxen 500 mg, twice daily; diclofenac, 100 mg twice daily; and piroxicam 20 mg/day), and acetaminophen 1000 mg twice daily. Treatment was provided for 6 months.

Direct medical costs and clinical effects of patients treated with any of the study treatment alternatives were estimated in order to identify the differences among them and to obtain an incremental cost-effectiveness ratio (ICER), integrating the values obtained from the study to the following formula (18,20):

$$ICER=\frac{Total\cos ts_{A}-Total\cos ts_{B}}{Effectiveness_{A}-Effectiveness_{B}}$$

Thus, ICER was obtained by dividing total net costs (incremental costs) between the total net effectiveness (incremental effectiveness) for two alternative treatments (A and B), in this case, two drugs (nonselective NSAIDs vs. celecoxib; acetaminophen vs. celecoxib).

Effectiveness measure used for this evaluation was the number of patients with pain control and no adverse events per each 1000 patients treated with any of the study alternatives.

### Transition

In order to identify data used to feed the proposed model, a qualitative systematic literature review was conducted with the following objectives: 1) to identify the probability of developing any of the possible clinical results (pain control or no pain control) after using one of the drugs proposed as alternatives for this evaluation for OA treatment, 2) to identify the probability of the occurrence of serious adverse events with any of the alternatives compared.

In this study, the QUORUM (quality of reporting of meta-analyses) recommendations were followed ; the aspects not mentioned were not realized. Search strategy planned for the systematic review was through electronic databases: Ovid-Medline, Elsevier-Science Direct, Proquest, Ebsco-E-Journal Services and Interscience. With the following key words: "randomized clinical trial", "arthrosis", "celecoxib", "naproxen", "diclofenac", "piroxicam", "acetaminophen", "response rate", "safety", "peptic ulcer", "minor bleed", "major bleed", "nephrotoxicity", "cardiovascular events" identified in any field only in clinical trials published between 1994 and 2004, in English or Spanish. For searching clinical results information, only randomized clinical trials where study intervention was "celecoxib", "naproxen", "diclofenac", "piroxicam" or "acetaminophen" in adult patients with OA and where results were reported as the percentage of patients with joint pain control, as well as description of rate of adverse events, were included. Only studies where the first treatment scheme for pain control was administered with one of the alternatives included in this investigation were considered.

Sixty clinical trials showing treatment efficacy and/or safety were identified. Due to the great variety of efficacy definitions, only those from studies with results expressed as clinical improvement, whether through a scale or with a percentage of joint pain improvement, were selected. Studies evaluated only knee and/or hip pain [28]. Adequate pain control was defined as a 50% change between baseline and results obtained after the administration of the study drug as shown by Ta et al. [29].

Only two studies with at least 12 weeks of follow-up were included, and it was assumed that pain control probability remained constant during the 6-month study period.

Celecoxib effectiveness was obtained in six clinical trials [29-34]. Two were compared vs. acetaminophen, three vs. naproxen and two vs. lumiracoxib, with sample sizes between 70 and 1684 patients. To identify the effectiveness of the nonselective NSAID group, the three studies evaluating naproxen compared to celecoxib were used and one clinical trial measuring piroxicam efficacy [33] was added, the latter compared to meloxicam. For acetaminophen effectiveness estimation, two clinical trials were identified, PACES (acetaminophen of celecoxib efficacy studies) [32] and another comparing to placebo and diclofenac [35]. Rate of adverse events was also reported in these studies.

Probabilities of gastric, renal, and cardiovascular adverse events, both for celecoxib and the nonselective NSAID groups, were obtained from two large studies, CLASS (Celecoxib Long-term Arthritis Safety Study) [36] and TARGET (Therapeutic Arthritis Research and Gastrointestinal Events Trial) [37,38].

Effectiveness and probabilities for adverse events were also supported based on several systematic reviews published during the period the study was conducted [39-41]. With all the information, efficacy data for joint pain control and the probabilities to develop severe adverse events were obtained (Tables 1 and 2) [29-41].

**Table 1:** Efficacy probability data for joint pain control in patients with OA

Drugs	Pain control	Presence of adverse events	References
Celecoxib	0.6540	0.567	[29-34]

Nonselective NSAIDs	0.6091	0.45	[29-31]

Acetaminophen	0.515	0.68	[32,35]

OA, osteoarthritis; NSAIDs, nonsteroidal anti-inflammatory drugs.

Effectiveness measure used for this evaluation was number of patients with pain control and no adverse events.

**Table 2:** Severe adverse event probabilities

Drugs	Peptic symptoms	GI bleeding	Adverse cardiovascular events	Nephrotoxicity
Celecoxib	0.50	0.0046	0.0039	0.0069

Nonselective NSAIDs	0.618	0.0136	0.0047	0.009

Acetaminophen	0.343	0	0	0

NSAIDs, nonsteroidal anti-inflammatory drugs.

References 29–35 were used for data.

The drug reported with the highest efficacy for the treatment of knee and/or hip OA is celecoxib, followed by any of the nonselective NSAIDs and, ultimately, acetaminophen.

### Use of resources and cost estimation

Patients attending the first level of health care are treated by specialists in family medicine or by general practitioners with several years of clinical experience. If a patient cannot control his/her symptoms, he/she is referred for evaluation by a specialist in a Hospital General de Zona (HGZ) which, in general, is a rheumatologist or an internist. Finally, and in more advanced stages of the disease, the patient is treated by an orthopedic surgeon in a third-level orthopedic-traumatology hospital. If serious adverse events occur, these are treated by different specialists: peptic symptoms by a gastroenterologist, GI bleeding as an acute event is treated by the emergency services of the HGZs and, if patients need to be hospitalized, by gastroenterologists and/or internists. In the case of adverse renal events, patients are treated by nephrologists at the HZG and, for cardiovascular events, by cardiologists from the second- and third-level health care institutions.

Identification of the resource use pattern was made through the description of a series of type cases, which describe the average patient in each of the tree branches (the three tree branches are based on type of drug used for treatment (Figure 1) and through the experts' opinion, the type of medical resources to be used for his/her medical care was obtained. The group of consensus experts was integrated by 18 family doctors, 5 gastroenterologists, 5 internists, 4 specialists in medical/surgical emergencies, 3 nephrologists, 3 cardiologists, 5 rheumatologists and 10 orthopedists working at the third level of health care. According to their specialty, all described the use of resources for patients with OA. This information was complemented with the review of clinical files to estimate costs for complications within the institutional setting *(n *= 120).

Unit costs for each resource used were identified in order to estimate an expected mean total cost. Estimation of the use of resources for patients not presenting adverse events was performed by family doctors, rheumatologists and orthopedists.

Estimation was done for the use of resources for medical care due to adverse events. In the case of GI events, specialists in medical/surgical emergencies, internists and gastroenterologists were interviewed. For nephrotoxicity treatment, nephrologists were consulted, and for the description of the resource use pattern in the case of cardiovascular events, cardiologists were interviewed. Each specialist must have proven that he/she was working at the IMSS with clinical experience of at least 5 years and certification issued by the corresponding specialty board. These physicians did not know the study hypothesis.

Information on the time of use, type and amount of drugs used, number and type of laboratory tests performed during ambulatory treatment and/or hospitalization, number of inter-consultations with other services, and number and type of surgical interventions were obtained for each case type.

Costs for each resource identified were obtained from several information sources. Unit prices for laboratory and imaging tests were identified through the Planning and Finance Department at the *Hospital de Traumatología y Ortopedia: Unidad Médica de Alta Especialidad "Dr. Victorio de la Fuente Narváez"*; moreover, IMSS official unit costs published in the *Diario Oficial *were identified [42]. Prices of drugs used in medical interventions at the IMSS were obtained from the Institute Web site [43].

### Time Horizon

Research time horizon was 6 months, similar to other studies [44,45]. During this time period intertemporal preferences of physicians and/or patients were not expected to change; thus, discount rates were not applied in the investigation.

### Sensitivity Analysis

A one-way sensitivity analysis was conducted to determine the minimum values needed to have the most cost-effective option to control joint pain. Sensitivity analysis also aimed to identify result robustness; thus, changes in some initial assumptions were made to observe if conclusions were maintained towards the same direction. Therefore, a probabilistic sensitivity analysis was conducted to introduce a certain level of uncertainty using a first-order Monte Carlo simulation that allowed the identification of potential variation both in costs and effectiveness and to observe their dispersion levels. Probabilistic sensitivity analyses used triangular distributions of costs (dispersion obtained from the hospital records) and effectiveness.

Finally, with the same simulation, the net economic benefits (NEB) analysis was conducted. NEB is an analysis that describes the uncertainty in the incremental effectiveness and cost values. Economic benefits may also be understood as the profits an institution may obtain for using a particular treatment. The NEB has the following formula:

$$NB_{A}=\mu_{E_{A}}*\lambda -\mu_{C_{A}}$$

where economic benefits for treatment A are obtained from the difference between mean effectiveness measure (μ_E_) multiplied by the willingness of the healthcare institution to pay (λ) and mean costs (μ_C_) for such alternative (44-46-48).

The project was carried out according to IMSS investigation regulations and was approved by the IMSS Health Coordination Ethics and Investigation Committee (No. 2005-785-142). In order to perform the economic assessment, the authors used the software Tree Age 2007 (Copyright^© ^1988–2007 by TreeAge Software, Inc. All rights reserved. Williamstown, MA).

## Results

### Costs

Health care costs for one patient in the first level of care during a period of six months is, on average, $2,388.59 Mexican pesos (MXP) [1 USD = 10.00 MXP (September 2008)]. This includes a consultation for diagnosis and three follow-up consultations, along with the following laboratory tests: hematology, C-reactive protein, rheumatoid factor, determination of uric acid concentration and one chest x-ray. A nonselective NSAID was prescribed and, if there was no response, acetaminophen was then added. When gastric symptoms were present, a histamine h-receptor antagonist such as ranitidine was added or the patient was referred to the second-level of care for evaluation. To keep simple, cost compounds are not specified for each procedure (adverse events).

In the case of second-level care during a 6-month treatment period for a patient with no adverse effects, the estimated cost was $2,165.15 MXP. This includes a diagnostic consultation by a rheumatologist, which implies one chest x-ray as well as three follow-up consultations and administration of initial treatment. In this case, acetaminophen is used and, if no response is achieved, a nonselective NSAID is prescribed.

**Figure 2:**
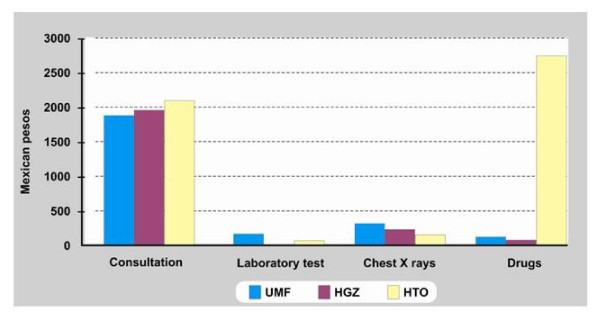
**Cost components for treating patients with OA at the Instituto Mexicano del Seguro Social.** UMF, Family Medicine Unit (Unidad de Medicina Familiar); HGZ: General Hospital (Hospital General de Zona); HTO: Orthopedic and Traumatology Hospital (Hospital de Traumatología y Ortopedia).

As far as third level health care concerns, health care costs for the 6-month period is, on average, $5,051.84 MXP (Figure 2). This includes two medical consultations, one chest X rays, determination of clotting time and urinalysis. Generally, patients are treated with a cyclooxygenase-2 inhibitor.

**Figure 3:**
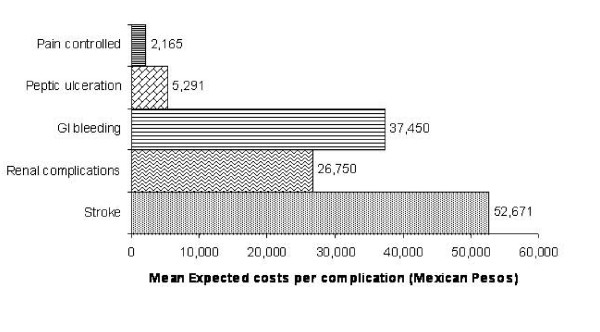
**Mean cost for OA treatment according to different scenarios and to each adverse event at the Instituto Mexicano del Seguro Social.** GI, gastrointestinal.

The highest cost generated for OA treatment was for medical consultation except for the third level where the cost of the drug is higher than the cost of medical consultations (Figure 2). When assessing adverse events, costs for treatment of peptic symptoms were $5,800.36 MXP during the 6-month study period. This included two medical consultations, one hematology test, one endoscopy, and continuous treatment with ranitidine and aluminum and magnesium gel (Figure 3).

The cost for GI bleeding associated with the use of drugs was $37,282.82 MXP. This included 1 day at the emergency service and 7 days, on average, of hospitalization as well as one or two endoscopies, hematology and blood chemistry. In addition, treatment is initiated with omeprazole administered IV and, later on, orally (Figure 3).

Medical care for a patient with serious renal damage was estimated at $26,998.66 MXP; this included 1 day at the emergency unit and, on average, 7 days of hospitalization, laboratory tests: blood chemistry, serum electrolytes, urinalysis, creatinine clearance, one ultrasound along with management with ASA, diuretics and antihypertensive agents such as angiotensin converter enzyme inhibitors (ACEI). For the case of cardiovascular events (CVE), data obtained for the IMSS came from the study conducted by Mould et al. [46]. Mean cost for myocardial infarction was $110,552.00 MXP and for cerebrovascular accident it was $52,671.00 MXP.

### Cost-effectiveness Analysis

The OA drug with the lowest cost, considering the possibility and treatment of adverse events, was celecoxib ($6,524.6 MXP/patient during 6 months of treatment), although differences are not that significant with the use of nonselective NSAIDs, but they are with the use of acetaminophen. As far as effectiveness is concerned, the drug with the largest number of patients with pain control without developing adverse events is again celecoxib, followed by nonselective NSAIDs and, ultimately, acetaminophen (Table 3). When integrating both measures (costs and effectiveness) within the deterministic analysis, it is observed that celecoxib is superior to the other two choices, with a lower cost and higher effectiveness.

**Table 3:** Incremental cost-effectiveness analysis (direct medical costs and clinical effects of patients treated with alternatives therapies)

**Treatments**	Costs*	**Costs ▲^†^**	**Effectiveness^‡^**	**Effectiveness▲**	ACER**	**ICER^††^**
Celecoxib	6,524.6		371		17.595	
Nonselective NSAIDs	6,587.4	62.8	274	97	24.033	Dominance
Acetaminophen	7,026.7	502.1	270	101	26.029	Dominance

*Estimated costs by patient (Mexican pesos).

^†^Incremental costs.

^‡^Number of patients with pain control without adverse events.

**Average cost-effectiveness ratio.

^††^Incremental cost-effectiveness ratio.

NSAIDs, nonsteroidal anti-inflammatory drugs.

### One-way sensitivity analysis

For this type of analysis, it was decided to hypothetically vary the effectiveness of the alternatives compared in this investigation in such a way that the best option (celecoxib) is no longer cost-effective (Tables 4 and 5).

**Table 4:** Probabilistic sensitivity analysis with first-order Monte Carlo simulation

**Drugs**	Mean ± SD*	**Median**	**Interquartile range**	
			5%	95%
Celecoxib				
Cost	6,198.7 ± 15,507.1	5,016.6	3,653.1	10,795.3
Effectiveness	365.0	315.0	125.0	452.0
Nonselective NSAIDs				
Cost	6,528.2 ± 6,199.9	5,300.0	2,340.7	14,497.9
Effectiveness	289.0	265.0	84.0	396.0
Acetaminophen				
Cost	6,994.3 ± 46,583.7	5,721.5	2,292.7	15,994.9
Effectiveness	275.0	259.0	74.0	351.0

*Standard deviation.

Costs are expressed in Mexican pesos and effectiveness is according to number of patients with pain control without adverse events.

NSAIDs, nonsteroidal anti-inflammatory drugs.

**Table 5:** One-way sensitivity analysis (minimum values in order to be the most cost-effective option to control joint pain)

		Type of dominance	
		Extended*	Absolute
Celecoxib			
Control joint pain	↓	44.0%	
Control joint pain without adverse events	↓	41.0%	
Nonselective NSAIDs			
Control joint pain	↑	67.5%	82.5%
Control joint pain without adverse events	↑	49.5%	63.0%
Acetaminophen			
Control joint pain	↑	48.0%	55.0%
Control joint pain without adverse events	↑	94.0%	it is not feasible

NSAIDs, nonsteroidal anti-inflammatory drugs.

Extended dominance is defined as the set of all possible mixed therapeutic strategies that dominates a unique strategy in both higher effectiveness and less cost [67].

In this case, celecoxib would have to decrease it effectiveness for joint pain treatment up to 44% and, for control with no adverse events, up to 41% in order not to be any longer the most cost-effective option of the compared alternatives. Nonspecific NSAIDs would have to increase their effectiveness up to 67.5% for pain control and up to 49.5% for pain control without the presence of adverse events to be the most cost-effective option using the definition of extended dominance. To be absolute, NSAIDs would have to increase their effectiveness up to 82.5% for pain control and 63.0% for pain control without the presence of adverse events (for extended dominance definition see Table 5). In the case of acetaminophen, it would need to obtain an absolute dominance just for pain control with an effectiveness of 55%, but for pain control with no adverse events its efficacy would have to increase up to 94%, but only to reach an extended dominance.

### Probabilistic sensitivity analysis

**Figure 4:**
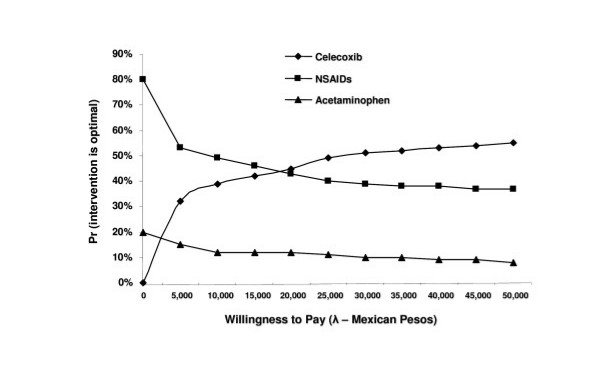
Cost-effectiveness acceptability curves for the decision concerning the most efficient management of OA at the Instituto Mexicano del Seguro Social.

For conducting the probabilistic sensitivity analysis, a hypothetical cohort of 10,000 samples using the first-order Monte Carlo method was previously simulated. With this simulation, it is expected to have a significant number of measures that allow estimation of the variability magnitude due to chance, both for costs and effectiveness. With this data, it is possible to construct acceptability curves for each treatment. These curves demonstrate the probability for a treatment to be cost-effective, depending on the willingness to pay by the healthcare institution. Figure 4 shows the acceptability curves for the three alternatives for joint pain due to OA. It may be observed in this plot how celecoxib is the most cost-effective option in 45% of cases, regardless of willingness to pay. Nonspecific NSAIDs are cost-effective at a 35% rate and acetaminophen at 20%.

**Figure 5:**
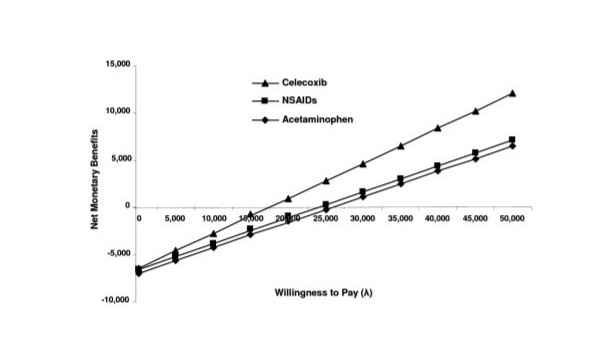
Net economic benefits for joint pain treatment due to OA at the Instituto Mexicano del Seguro Social.

When estimating the NEB, it is observed that higher savings for the institution may be obtained with celecoxib, regardless of willingness to pay (Figure 5), followed by nonspecific NSAID treatment and similarly with acetaminophen. There are no significant differences between the latter two drugs.

In conclusion, the sensitivity analysis stated that, regarding the one-way analysis, celecoxib would be the most cost-effective option unless it has a marked decrease in its effectiveness or the other alternatives would have to significantly increase their effectiveness. In the probabilistic analyses, both in the construction of the acceptability curves and in the estimation of NEBs, celecoxib will remain as the most cost-effective option compared to the other two alternatives for the treatment of joint pain due to OA of the knee and/or hip at the IMSS.

## Discussion

Through this cost-effectiveness analysis, it has been shown that celecoxib was superior to nonspecific NSAIDs and acetaminophen. There was a lower use of resources with this type of treatment, especially due to a lower rate of adverse events, resulting in a decrease in health care costs. Such results did not change when a probabilistic sensitivity analysis was performed. Thus, currently it may be considered that this is the best treatment alternative for the IMSS.

This investigation conducted an economic evaluation considering both health results and the cost for medical care associated with the use of cyclooxygenase-2 inhibitors, nonspecific NSAIDs, and acetaminophen. In the case of the effectiveness analysis, through the systematic review, some important differences were found, especially when defining an effectiveness measure as pain control with no adverse events. In other economical evaluation models, it was assumed that pain control effectiveness was the same and that there were differences only in the frequency of adverse events [47-49]. In this study we were more specific with the effectiveness measure, making the differences among drugs more evident.

In the present research the pain control without adverse events in OA patients was used as an effectiveness measure. In addition, it is important to mention that all the clinical trials which include NSAIDs, acetaminophen and celecoxib in the management of OA are mainly focus in pain control [13,45,50-52]; In this sense, this economic evaluation is in line with the effectiveness measure common used in the literature. Nevertheless, our assessment is leaving out what other authors employed as a complete evaluation for the treatment of OA which include the pain control but also the affected articulation functions [53].

When conducting the complete cost-effectiveness analysis, the results of this study are similar to other models published [44,47-49] where the use of the drug from the cyclooxygenase-2 inhibitors group is the most cost-effective. An important issue to be mentioned is that this model included the probability to develop, within the 6 months of treatment, cardiovascular and nephrotoxicity events associated with non-specific NSAIDs, which is different from other models where only gastrointestinal events (peptic and/or digestive tract bleeding) were considered [45]. The studies encouraging the launching of rofecoxib into the market based their rationale mainly on the presence of cardiovascular events [54-57]. In the case of celecoxib, very low frequencies for the development of this type of event during the time specified for this study were estimated (in the systematic review); in fact, they are similar for the non-specific NSAIDs group, which is consistent with the recent FDA recommendations [58] on the use of these types of drugs during short periods of time. A recent systematic review confirms this assumption, where the RR for cardiovascular events with celecoxib was 1.06 (95% CI 0.91–1.23) and with naproxen it was 0.97 (95% CI 0.87–1.07) [54]. A meta-analysis showed similar results with an RR for vascular events of 1.60 (95% CI 0.90–2.9) with celecoxib and 0.92 (95% CI 0.67–1.26) for naproxen [13]. Another meta-analysis, which shows only non-specific NSAIDs findings, reported an RR for acute myocardial infarction of 0.99 (95% CI 0.88–1.11) with naproxen, and they did not evaluate celecoxib. It has been mentioned that the risk for cardiovascular events is similar between celecoxib and naproxen [50].

In a meta-analysis of clinical trials published up to June 2006, including 37 studies evaluating celecoxib, a RR of 0.61 (95% CI 0.40–0.94) for renal impairment and 0.83 (95% CI 0.71–0.97) for hypertension was shown [9]. In another study published at the end of 2007, a RR for acute renal impairment of 2.00 (95% CI 1.32–3.04) and 1.33 (95% CI 0.94–1.88) for celecoxib at doses >200 mg/day and <200 mg/day, respectively, was reported. With the use of naproxen, a RR of 3.62 (95% CI 2.01–6.53) and 1.65 (95% CI 0.88–3.08) at a dose >750 mg/day and <750 mg/day, respectively, was reported. This confirmed that the drug with the lowest risk was celecoxib, especially at a dose of 200 mg/day or less [6]. In a recent study it was confirmed that the drug that had less risk to suffer from a hospitalization for gastrointestinal bleeding was the celecoxib [59]

One limitation of this study is that the systematic review included only articles published; thus, a possibility of a publication bias cannot be ruled out. On the other hand, the reports of the clinical trials included in the study do not necessarily reflect the results that would be obtained in the Mexican population because other factors modifying local clinical response may exist. The probabilities feeding the model were obtained from publications from 1994 to 2004, probabilities that may have changed with current data.

Regarding the estimations for the use of resources, they represent only local clinical practices; thus, external validity would be compromised only to similar practices. Nevertheless, it may be stated that this model is a good approximation and provides an idea of what would happen in the reality of the IMSS, and other health care systems in developing countries.

Regarding the time horizon, this assessment is in line with the reported timelines used by other researchers in the literature, specially the time needed to control the articular pain [45]; it is honest to say that with the timeline employed in our study it was not feasible to assess neither compliance nor long-term adverse events. Although, healthcare costs estimations and presence of side effects have been reported mainly for this time horizon within the literature [45].

OA is a disease with a trend towards a high prevalence [1,514, [60-62]] because it is strongly associated with aging, among other factors. With the demographic and epidemiological transition [63] in developing countries, health systems face a high demand for medical services [26,64]. Within a low-resource environment, it is important for decision makers to efficiently choose the use of available resources, a reason why economic evaluation analyses are important when making the selections [22,23,26].

According to our knowledge, this is the first analysis of this type carried out in a developing country. IMSS is a prototype for health institutions that aim to provide social security to the population and shares many common characteristics with other social security institutions in other developing countries [65]. In developing countries, it has been considered that the priority for investigation is the one allowing decision makers to more clearly understand how health care resources should be used [66]. This type of study provides an answer to this need, and it can be pointed out that, in countries where the social security system is distributed such as the IMSS and organized according to the levels of care previously mentioned, results may be extrapolated to them.

## Conclusion

The least expensive drug for the treatment of knee and/or hip OA, considering the possibility and treatment for adverse events, was celecoxib ($6,524.6 MXP per patient during 6 months of treatment), in addition to it being the most effective treatment.

With this analysis, it may be stated that the use of cyclooxygenase-2 inhibitors, such as celecoxib, is the treatment with the best cost-effective results (lowest mean cost-effectiveness ratio $17.5 MXP/patient), which may result in a lower use of resources due to the presence of adverse events related to drugs for treating OA and the consequent cost decrease.

Within the sensitivity analysis, it may be pointed out that regarding the univariate analysis, celecoxib would have to markedly decrease its effectiveness, or the other alternatives would have to increase significantly. On the other hand, in the probabilistic analysis, both in the construction of the acceptability curves and in the estimation of net economic benefits, the most cost-effective option remains to be celecoxib, compared to the other two alternatives for the treatment of knee and/or hip joint pain at IMSS.

## Abbreviations

QALY: quality-adjusted life years; ICER: incremental cost-effectiveness ratio; PACES: acetaminophen of celecoxib efficacy studies; NSAIDs: nonsteroidal anti-inflammatory drugs; CLASS: Celecoxib Long-term Arthritis Safety Study; TARGET: Therapeutic Arthritis Research and Gastrointestinal Events Trial; IMSS: Instituto Mexicano del Seguro Social; COX: cyclooxygenase enzyme; RR: relative risk; CI: confidence interval; FDA: Food and Drug Administration; ACEI: angiotensin converter enzyme inhibitor; CVE: cardiovascular events; NEB: net economic benefits; HGZ: Hospital General de Zona.

## Competing interests

This study received financial support from Pfizer Laboratories; however, both in planning and in the interpretation of results, this company remained blinded to the manner that this study was conducted and analyzed. The project was carried out according to IMSS investigation regulations and approved by the IMSS Health Coordination Ethics and Investigation Committee (No. 2005-785-142). This obligated investigators to present detailed reports of the study every 6 months. ICH and JFMQ received financial support as they were Pfizer study supervisors. JFMQ, during the study, was an investigator working at the Unidad de Investigación en Economía de la Salud, Instituto Mexicano del Seguro Social. He is currently Outcomes Research Manager for Pfizer. JMMA was an advisor who was paid for this study and received funds from Pfizer for the development of this manuscript. RTG, MVGR, RLPD and JGE declare that they have no competing interests.

## Authors' contributions

ICH, JFMQ and JGE, conceived, designed, analyzed and interpreted the study. RTG, MVGR, RLPD and SSG contributed in the systematic review of literature, in the interviews with medical staff, and in the interpretation of the results. JMMA participated in data analysis and interpretation, as well as in writing the initial and final manuscript of this study. All authors read and approved the final manuscript.
